# Profound Hypothermia as a Rare Presentation of Uremic Syndrome in a Patient With Advanced Chronic Kidney Disease: A Case Report and Literature Review

**DOI:** 10.7759/cureus.103418

**Published:** 2026-02-11

**Authors:** Fahmi A Aldhaheri, Abdulrahman Bahatheq, Rowaid S Yazbik, Raghda Alawadi

**Affiliations:** 1 Nephrology, Dr. Soliman Fakeeh Hospital, Jeddah, SAU; 2 Internal Medicine, Dr. Soliman Fakeeh Hospital, Jeddah, SAU

**Keywords:** chronic kidney disease, end stage kidney disease, hemodialysis, hypothermia, uremic syndrome

## Abstract

Hypothermia is a rare but serious manifestation of uremic syndrome, which is rarely reported in the literature in patients with advanced chronic kidney disease (CKD). Hypothermia, defined as a core body temperature below 35°C, is categorized into mild (32-35°C), moderate (28-32°C), and severe (<28°C) stages, each associated with increasing risk of mortality. The human body relies on enzymatic processes and thermoregulatory mechanisms to maintain homeostasis, which can be disrupted in uremia due to toxin accumulation from impaired renal function. We present a case of a patient with CKD stage V who was planned for dialysis initiation but developed clinical signs of uremia before, in the form of uremic hypothermia with a core temperature of 34°C. Other potential causes, such as sepsis, trauma, exposure, and endocrine abnormalities, were ruled out. Pathophysiology may involve the accumulation of endogenous cryogens leading to peripheral vasodilation and suppressed shivering, thus impairing thermoregulation. Remarkably, the patient's symptoms and temperature improved following the initiation of dialysis, supporting a possible association between uremia and hypothermia. This case highlights profound hypothermia as a rare and potentially life-threatening presentation of uremic syndrome. Prompt recognition and initiation of renal replacement therapy are crucial for recovery.

## Introduction

Maintenance of core body temperature within a narrow physiological range is essential for normal cellular metabolism and integrated organ function. Hypothermia, defined as a core temperature below 35°C, is associated with progressive physiological derangements and is conventionally classified as mild, moderate, or severe. As core temperature declines, the risk of morbidity and mortality increases substantially. Moderate to severe hypothermia may result in multisystem dysfunction, particularly affecting the cardiovascular, respiratory, and nervous systems, with complications including hypotension, bradycardia, ventricular arrhythmias (notably ventricular fibrillation), coma, areflexia, and, in advanced cases, asystole [1].

Although hypothermia is most frequently attributed to environmental cold exposure, non-environmental etiologies are increasingly recognized in hospitalized and chronically ill populations. These include sepsis, trauma, central nervous system pathology, endocrine disorders such as hypothyroidism and adrenal insufficiency, malnutrition, drug intoxication, and metabolic disturbances [2]. Accurate identification of the underlying cause is critical, as management extends beyond rewarming to targeted treatment of the precipitating condition and mitigation of recurrent or fatal complications.

Uremic syndrome represents an underrecognized yet clinically relevant cause of hypothermia in patients with advanced chronic kidney disease (CKD). Uremia is characterized by the accumulation of nitrogenous waste products, including blood urea nitrogen and creatinine, in addition to a broad spectrum of retained solutes that disrupt enzymatic, metabolic, and neurohumoral homeostasis [3]. These toxins contribute to the systemic manifestations of uremia, including encephalopathy, platelet dysfunction, autonomic dysregulation, and impaired thermoregulation.

The mechanisms underlying uremic hypothermia are likely multifactorial. Experimental and clinical data suggest that uremia may attenuate hypothalamic thermoregulatory responses, reduce basal metabolic rates, and impair heat-conserving mechanisms such as peripheral vasoconstriction and shivering [4,5]. Furthermore, the accumulation of endogenous cryogenic substances has been proposed to directly suppress thermogenic pathways. Uremic hypothermia was documented in human dialysis patients with an increase in temperature after correction of uremia and in dogs after urea injections and bilateral ureteral ligation [6]. Importantly, resolution of hypothermia after initiation of dialysis has been reported, reinforcing a causal association between uremia and disordered thermoregulation [7].

Despite these observations, uremic hypothermia remains infrequently recognized and underrepresented in contemporary clinical literature [8]. Failure to identify uremia as the underlying etiology of unexplained hypothermia may delay initiation of renal replacement therapy and increase the risk of adverse outcomes. This case underscores uremic hypothermia as a clinically significant but often overlooked manifestation of advanced CKD and highlights the importance of maintaining a high index of suspicion in patients presenting with unexplained hypothermia.

## Case presentation

A 60-year-old male patient with a history of CKD stage V, heart failure with preserved ejection fraction (HFpEF), hypertension (HTN), type 2 diabetes mellitus (DM), and previous cerebrovascular accident (CVA) complicated by secondary epilepsy was admitted to our hospital in January 2025. Initially, he was managed under the cardiology service for hypertensive pulmonary edema. During hospitalization, his renal function deteriorated, prompting a referral to the nephrology service.

Over the next several days, the patient’s renal function worsened, with his creatinine levels increasing from 4.5 mg/dL to 7.0 mg/dL, and blood urea nitrogen (BUN) rising to 144 mg/dL by January 22. The patient developed classic uremic symptoms, including slurred speech, nausea, vomiting, and pruritus affecting both lower limbs.

Physical examination revealed abdominal distention, clear lung fields, and grade 1+ pitting edema in both lower limbs. No bradycardia and ECG showed sinus tachycardia of 109 bpm.

**Figure 1:**
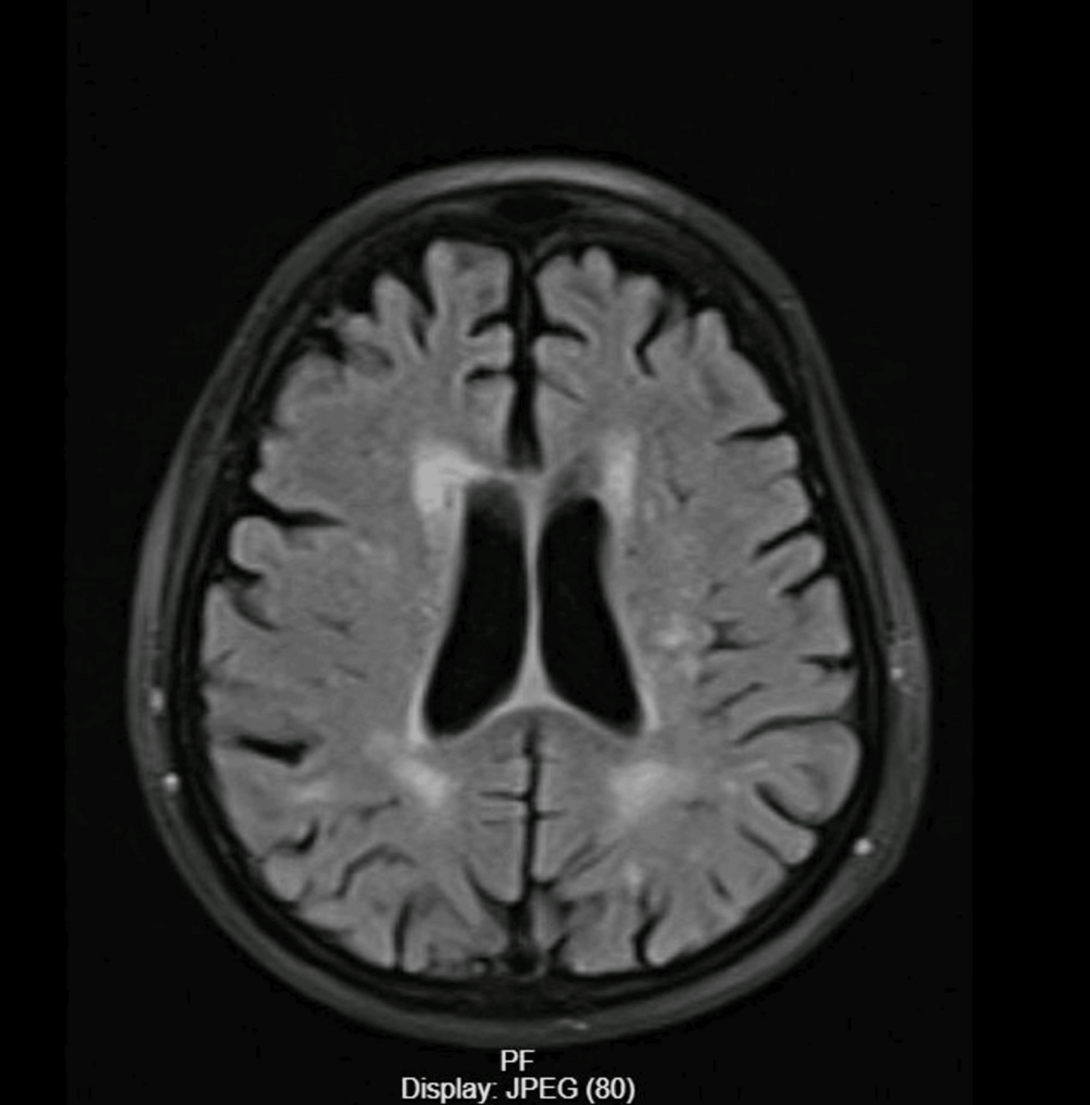
MRI brain The image shows dilated cerebral ventricles with no midline shift or deformity, bilateral periventricular deep and subcortical white matter, and patchy areas of high FLAIR WI signals. The picture is impressive of chronic arteriolosclerotic leukoencephalopathy. FLAIR: fluid-attenuated inversion recovery; WI: weighted image

Given the progression of his kidney disease, dialysis initiation was decided. However, prior to dialysis, the patient exhibited a sudden drop in consciousness and a body temperature of 34°C (measured orally). CT brain and MRI brain were performed and were negative for acute brain insult (Figure 1).

A full metabolic profile showed no significant abnormalities (Table 1), and septic workup (Table 2) was negative. 

**Table 1:** Labs pre- and post-dialysis with reference ranges based upon hospital lab range BUN: blood urea nitrogen; TSH: thyroid-stimulating hormone

Labs	Pre-dialysis	Post-dialysis	Reference range
Serum creatinine	7.0	3.6	0.67-1.17 (mg/dl)
BUN	144	70	6-20 (mg/dl)
Serum potassium	4.9	3.6	3.5-5.1 (mmol/L)
Serum sodium	133	139	136-145 (mmol/L)
Calcium	7.7	8.8	8.6-10 (mg/dl)
Phosphorus	8.5	3.7	2.5-4.5 (mg/dl)
Magnesium	2.4	2.2	1.6-2.6 (mg/dl)
Albumin	3.35	3.7	3.97-4.94 (g/dl)
Seum bicarbonate	20	25	22-29 (mmol/L)
TSH	1.1	-	0.27-4.2 (ulU/ml)
Random cortisol	47	-	3.7-19.4 (ug/dl)
Random glucose	163	181	80-120 (mg/dl)

**Table 2:** Septic workup RSV: respiratory syncytial virus

Cultures	Results
Peripheral blood cultures	Negative
Urine culture	Negative
Permanent dialysis catheter culture	Negative
Rapid PCR for (Flu A, Flu B, RSV, and SARS-CoV-2)	Negative

Hemodialysis was promptly initiated with incremental hemodialysis of low blood flow at 200 ml/min to prevent the occurrence of dialysis disequilibrium syndrome along with external warming techniques. The dialysate temperature was set at 37℃. The hypothermia was resolved within hours. No further hypothermic episodes were observed, and the other uremic symptoms, including the conscious level, were resolved with the incremental hemodialysis. The patient was discharged on maintenance hemodialysis three times per week.

**Figure 2:**
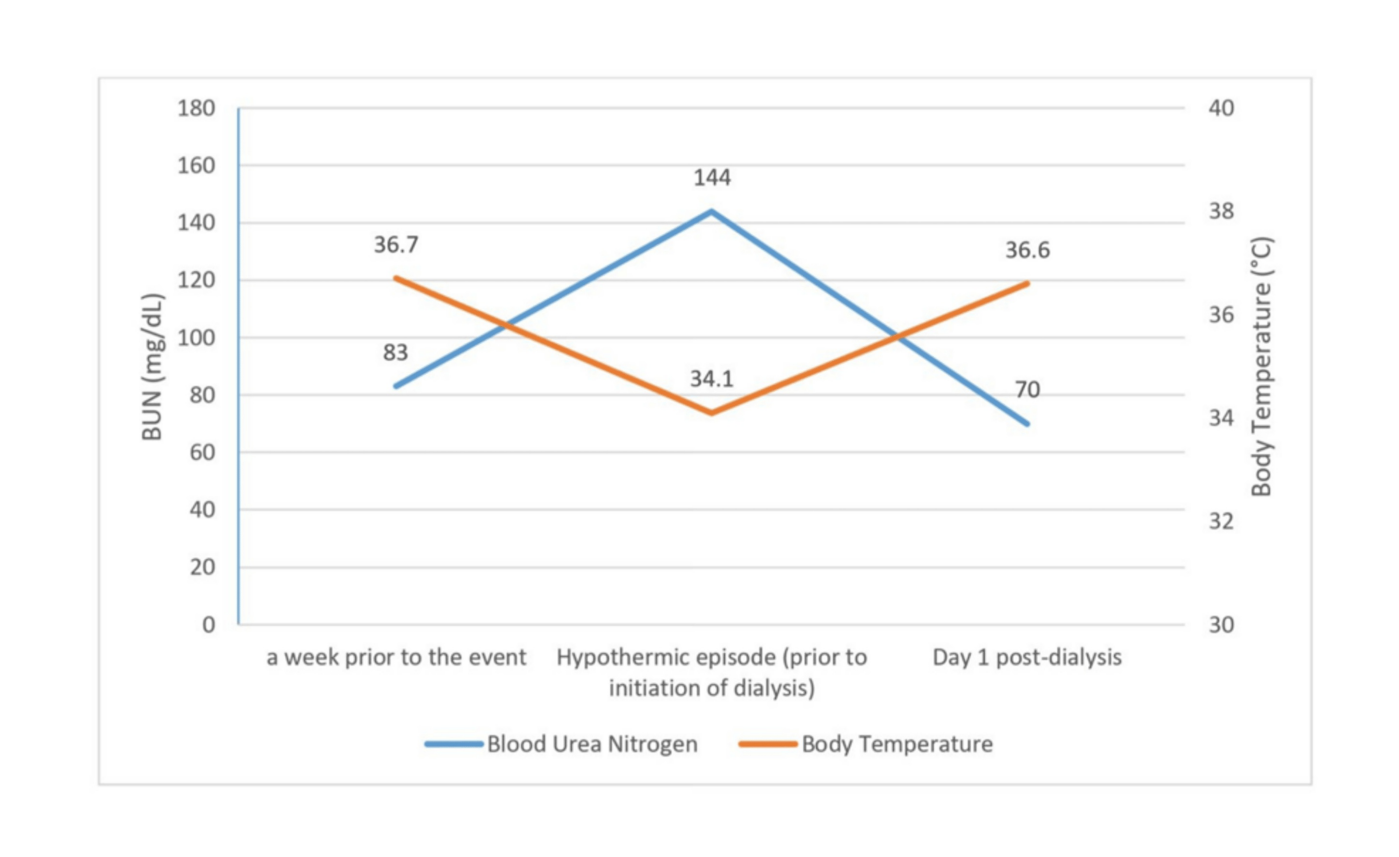
Clinical timeline illustrating the correlation between BUN levels and body temperature The graph highlights the peak of uremia (144 mg/dL) corresponding with the nadir of the hypothermic episode (34.1°C), followed by clinical stabilization on day 1 post-dialysis. BUN: blood urea nitrogen

The trend shows a progressive rise in BUN reaching its peak prior to dialysis initiation, followed by a marked decline post dialysis. Simultaneously, the body temperature showed a transient drop, then returned to baseline after dialysis (Figure 2).

## Discussion

Hypothermia is defined as a core body temperature below 35°C, which may result from environmental exposure or be secondary to underlying conditions such as sepsis, trauma, and endocrine disorders like hypoglycemia, diabetic ketoacidosis (DKA), adrenal insufficiency, and hypothyroidism [1].

Returning to our patient, known to have CKD stage V. During his hospital stay, his renal function worsened, with rising of his BUN to 144 mg/dL. The patient developed classic uremic symptoms, including nausea, vomiting, and pruritus.

The patient was planned for the initiation of hemodialysis (HD). However, his level of consciousness suddenly deteriorated, and he was found to have hypothermia. After the initiation of HD, his core temperature returned to normal levels, and he no longer experienced hypothermia.

The most common causes of hypothermia were ruled out, as the patient had normal thyroid function, cortisol levels, and blood sugar, and sepsis was excluded. The only abnormal lab result revealed is azotemia, where there is an elevated concentration of nitrogenous waste products, BUN, and creatinine.

This case demonstrates how profound hypothermia can be, presenting symptoms of uremic encephalopathy, particularly in advanced CKD. Early recognition and dialysis are crucial for recovery. In our case, no alternative cause was identified, and the resolution of hypothermia following dialysis supports uremia as the primary etiology.

The pathophysiology behind uremic hypothermia remains incompletely understood. Traditionally, it is thought to result from the generalized buildup of uremic solutes that interfere with thermoregulation. However, specific molecules such as cyanate may exert direct toxic effects on cellular metabolism [9]. These findings suggest that impaired cellular oxygen utilization and disrupted metabolic function in uremia, driven by the accumulation of highly osmolar solutes such as urea, may compromise transmembrane chemical gradients and mitochondrial respiration, resulting in reduced energy production and heat generation [10].

Current literature on the association between uremia and hypothermia in humans is limited. In 1981, Kluger et al. performed an experimental study in rabbits to investigate the thermoregulatory effects of an endogenous cryogen [11]. They observed that injection of human urine resulted in a decrease in body temperature, mediated by peripheral vasodilation in a thermoneutral environment and by suppression of shivering without vasodilation in a cold environment. The authors further proposed that endogenous cryogens present in urine may modulate the thermoregulatory set point.

In 1987, Hohegenner et al. conducted an in vitro study in rats, suggesting that uremic hypothermia may arise from a decreased metabolic rate, potentially compounded by the direct cellular effects of toxic substances [12]. However, experimental models that reproduce uremic signs and symptoms by increasing solute concentrations in animals or humans remain scarce. Notably, hemodialysis has been associated with the resolution of hypothermia in uremic cats and dogs [6]. One case report described severe hypothermia in a patient with CKD Stage IV secondary to hypertension-induced renovascular disease, which improved after the initiation of dialysis [8].

The mechanism of hypothermia is thought to be due to the accumulation of endogenous cryogens in uremic syndrome, which may induce peripheral vasodilation and suppress shivering metabolism, thus leading to a drop in body temperature. Other potential explanations could be related to altered hypothalamic function [8].

## Conclusions

This case highlights a rare but important presentation of advanced chronic kidney disease in which profound hypothermia was the initial manifestation of uremic syndrome. It serves as a reminder that uremic hypothermia should be considered when patients with renal failure are present with unexplained hypothermia, particularly when common environmental or systemic causes are absent. Early recognition of this association can prevent unnecessary diagnostic testing and avoid delays in definitive treatment. The patient’s clinical improvement after initiation of dialysis underscores the effectiveness of renal replacement therapy in reversing uremic manifestations and restoring physiological stability. Overall, this case reinforces the need for clinical vigilance and timely intervention when evaluating hypothermia in patients with advanced kidney disease.
